# Ectonucleotidases in Intestinal and Hepatic Inflammation

**DOI:** 10.3389/fimmu.2019.00507

**Published:** 2019-03-19

**Authors:** Marta Vuerich, Simon C. Robson, Maria Serena Longhi

**Affiliations:** ^1^Department of Anesthesia, Beth Israel Deaconess Medical Center, Harvard Medical School, Boston, MA, United States; ^2^Division of Gastroenterology, Department of Medicine, Beth Israel Deaconess Medical Center, Harvard Medical School, Boston, MA, United States

**Keywords:** ectonucleotidase, ATP, adenosine, T-cell, intestine, liver

## Abstract

Purinergic signaling modulates systemic and local inflammatory responses. Extracellular nucleotides, including eATP, promote inflammation, at least in part via the inflammasome upon engagement of P2 purinergic receptors. In contrast, adenosine generated during eATP phosphohydrolysis by ectonucleotidases, triggers immunosuppressive/anti-inflammatory pathways. Mounting evidence supports the role of ectonucleotidases, especially ENTPD1/CD39 and CD73, in the control of several inflammatory conditions, ranging from infectious disease, organ fibrosis to oncogenesis. Our experimental data generated over the years have indicated both CD39 and CD73 serve as pivotal regulators of intestinal and hepatic inflammation. In this context, immune cell responses are regulated by the balance between eATP and adenosine, potentially impacting disease outcomes as in gastrointestinal infection, inflammatory bowel disease, ischemia reperfusion injury of the bowel and liver, autoimmune or viral hepatitis and other inflammatory conditions, such as cancer. In this review, we report the most recent discoveries on the role of ENTPD1/CD39, CD73, and other ectonucleotidases in the regulation of intestinal and hepatic inflammation. We discuss the present knowledge, highlight the most intriguing and promising experimental data and comment on important aspects that still need to be addressed to develop purinergic-based therapies for these important illnesses.

## Introduction

The “purinergic signaling hypothesis” dates back to 1972 when Geoffrey Burnstock discovered that eATP and derivatives modulate gut and urinary tract neurotransmission (1). Nucleotides modulate cell responses upon binding to purinergic receptors (2–6) and also provide mediators after ectonucleotidase-mediated hydrolysis into adenosine (7, 8). Adenosine regulates cellular immune responses upon binding P1 adenosine receptors (3, 9, 10).

Ectonucleotidases include ecto-nucleoside triphosphate diphosphohydrolases (ENTPDases), ecto-5′-nucleotidase (NT5E)/CD73, ecto-nucleotide pyrophosphate phosphodiesterases (E-NPPs); CD38/NADase; NAD glycohydrolases; nucleoside diphosphate kinase; ecto-F1-F0 ATP synthases (11) and adenylate kinases. ENTPDase1, 2, 3, and 8 are surface-located enzymes that hydrolyze ATP/ADP into AMP. ENTPD2 however displays preferential ecto-ATPase activity (12). NTPDases4,5, 6, and 7 are intracellular proteins, with ENTPD5 and 6 being secreted upon heterologous expression.

NT5E/CD73, which converts AMP into adenosine, has been described both as GPI-anchored protein or soluble enzyme (13, 14). Soluble CD73 (sCD73) mainly derives from shedding of lymphocytes (13) and is present in both serum and cell-free lymph of healthy individuals (15). Increase in sCD73 levels has been reported in inflammatory conditions (16) and was negatively correlated with disease severity in patients with acute pancreatitis (17).

While being constitutively present on different immune cells, ENTPD1/CD39 and NT5E/CD73 can be further induced upon exposure to oxidative stress and hypoxia, stimulation with pro-inflammatory cytokines or following aryl hydrocarbon receptor (AhR) engagement (4, 18–21).

In this review, we focus on the role of ENTPD1/CD39 and NT5E/CD73 in gastrointestinal and hepatic inflammation.

## Ectonucleotidases in Acute and Chronic GI Illness

**Figure 1:**
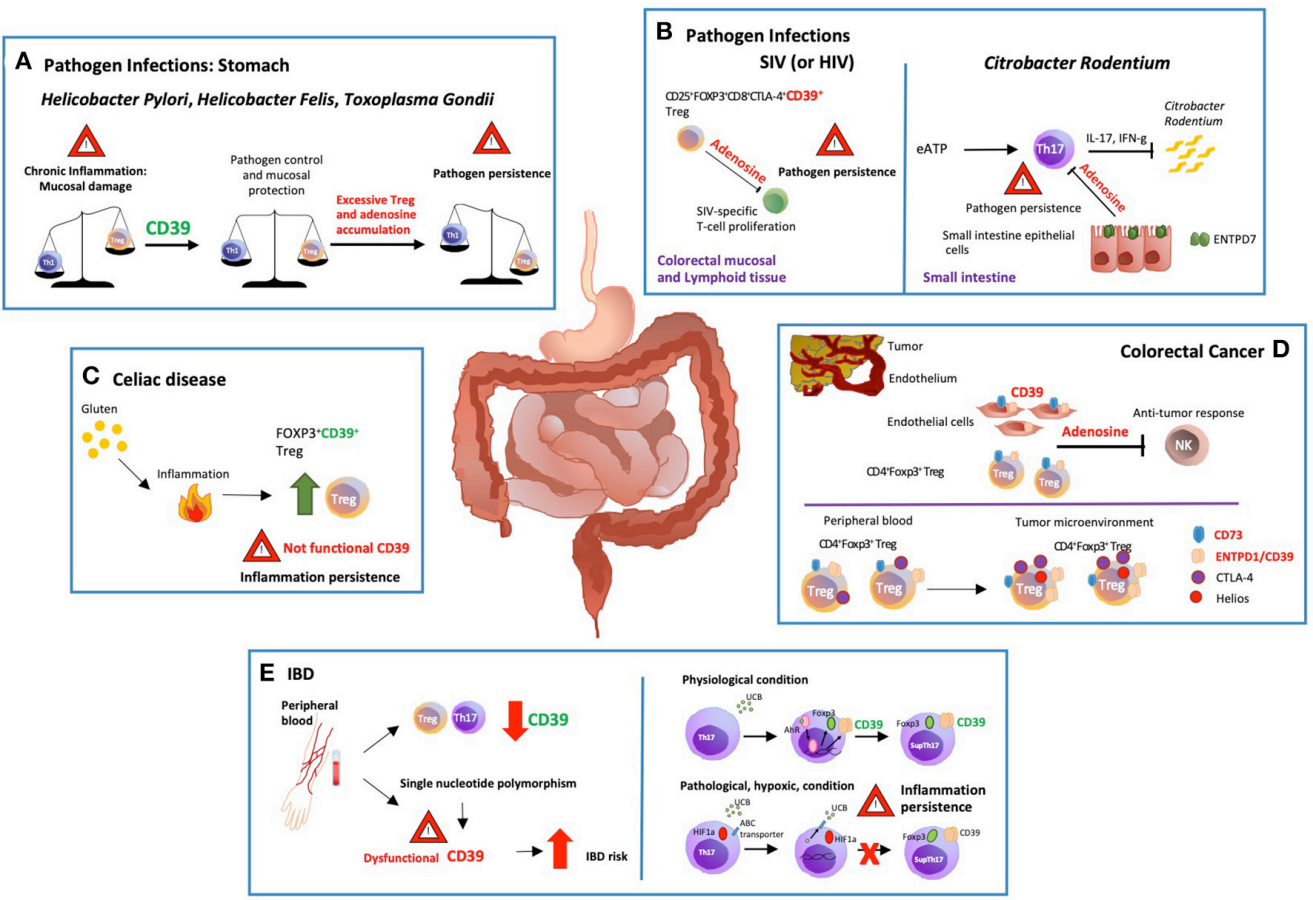
Modulation of gastro-intestinal disease by ectonucleotidases. **(A)** There are potent immune responses to gastrointestinal bacterial and parasitic infections e.g., *Helicobacter pylori, Helicobacter felis* and *Toxoplasma gondii*. The balance between pro and anti-inflammatory signals controls the development and outcome of the disease. Protracted release of Th1-related cytokines contributes to the establishment of chronic inflammation that might ultimately result in peptic ulcer disease and gastric cancer. ENTPD1/CD39 expression by regulatory T-cells (Tregs) modulates Th-cell responses; however, excessive immune regulation can also lead to pathogen persistence. **(B)** In a macacus rhesus model of pathogenic simian immunodeficiency virus (SIV) infection, there is rapid expansion of CD25^+^FOXP3^+^CD8^+^CTLA-4^+^CD39^+^ Tregs, especially in the colorectal mucosal and lymphoid tissues. This event limits anti-viral responses by suppressing the proliferation of SIV-specific T-cells. Treg accumulation is also observed in HIV patients, implicating that therapeutic strategies controlling the expansion of CD25^+^FOXP3^+^CD8^+^CTLA-4^+^ CD39^+^ Tregs might effectively control HIV infection restoring the anti-viral response. **(C)** Celiac disease is a chronic inflammatory disorder triggered by aberrant immune responses to dietary gluten. Exposure to gluten induces protective accumulation of FOXP3^+^CD39^+^ Tregs that, however, display defective suppressive function, and do not adequately control aberrant inflammatory responses. **(D)** ENTPD1/CD39 and CD73 are the dominant ectonucleotidases expressed by tumor endothelial cells and Tregs. Extracellular adenosine generated by CD39^+^ Tregs isolated from the blood of cancer patients inhibits and suppresses anti-tumor responses. Further, the tumor microenvironment impacts the phenotype and function of local cells, substantially limiting immunotherapeutic strategies. In this regard, most of the colorectal cancer-infiltrating Tregs are Helios^+^ and express higher levels of ENTPD1/CD39 and cytotoxic T-lymphocyte antigen 4 (CTLA-4), when compared to peripheral blood and colon-derived counterparts. **(E)** Low levels of ENTPD1/CD39 expression by Tregs and Th17-cells are observed in the peripheral blood of patients with inflammatory bowel disease (IBD). Moreover, single nucleotide polymorphisms associated with low *ENTPD1/CD39* mRNA levels, increase susceptibility to the disease. On the other hand, *in vitro* exposure to unconjugated bilirubin (UCB) results in increased levels of ENTPD1/CD39 and FOXP3 expression in Th17-cells derived from healthy individuals, through a mechanism mediated by aryl hydrocarbon receptor (AhR). However, Crohn's-derived Th17-cells remain refractory to UCB immunoregulation due to altered responses to hypoxia that inhibits AhR signaling by inducing ATP-binding cassette (ABC) transporters that promote UCB efflux out of Th17-cells.

Aberrant immune responses in gastric and intestinal disease might result in the development of chronic and progressive inflammatory statuses. In this setting, several studies emphasize the crucial role of the purinergic signaling in the modulation of GI conditions (Figure 1) (22).

### Gastrointestinal Infections

T-helper-cells are pivotal players in anti-bacterial responses (23). Protracted release of Th1-related cytokines, however, contributes to chronic inflammation that might ultimately result in peptic ulcer disease and gastric cancer, as in the context of *Helicobacter (H.) pylori* infection. However, inadequate Th1 immunity can lead to persistent infection as result of regulatory T-cell (Treg) accumulation (24–26) that supports pathogen persistence. Adenosine generation by ENTPD1/CD39 and CD73 on Treg and memory T-cells, strongly inhibits effector T-cell immunity (8, 27), as shown *in vitro* and in experimental models of *H. felis*-induced gastritis *in vivo* (28).

*Cd73*^−/−^ mice develop a more severe gastritis, associated with heightened levels of pro-inflammatory cytokines and impaired Treg function (28). Administration of an A2A adenosine receptor (A2AR) agonist to *Il-10*^−/−^ and Helicobacter-bearing mice attenuates gastritis lowering TNF-α and IFN-γ levels (29).

A comparable regulatory pathway has been observed in murine models of intestinal (and systemic) *Toxoplasma gondii* (*T. gondii*) infection. In the intestine of naive mice, conventional CD4^+^ T-cells and Tregs express both ENTPD1/CD39 and CD73. During acute *T. gondii* infection, CD73 expression is downregulated, with consequent diminished generation of immunosuppressive adenosine. As levels of the type-1 purinergic adenosine receptors are maintained, administration of receptor agonists ameliorates disease symptoms and associated dysbiosis (30).

The key role of ENTPD1/CD39 in the modulation of cellular immune response in the intestine has been suggested in a macacus rhesus model of simian immunodeficiency virus (SIV) infection. Infection with SIV results in rapid expansion of CD25^+^FOXP3^+^CD8^+^CTLA-4^+^CD39^+^ Tregs, especially in colorectal mucosal and lymphoid tissues, the preferential sites of virus replication. This development limits anti-viral responses by suppressing the proliferation of SIV-specific T-cells. Treg accumulation is also observed in HIV patients, implicating that therapeutic strategies aimed at containing Treg expansion might improve the control over HIV by restoring anti-viral responses (31, 32).

Purinergic signaling regulates also Th17-cell immunity (33). ENTPD7 expression in the epithelial cells of small intestine controls luminal ATP levels, therefore regulating Th17-cell development (34). In this regard, high ATP levels and Th17-cell accumulation are noted in the lamina propria of *Entpd7*^−/−^ mice and homeostasis can be restored by oral administration of ATP antagonists or antibiotics (34). In the absence of ENTPD7, commensal microbiota-dependent eATP release supports Th17-cell development (34). Accordingly, *Entpd7*^−/−^ mice are resistant to *Citrobacter rodentium* infection although suffering from severe experimental autoimmune encephalomyelitis, resulting from accumulation of IL-17 and IFN-γ (34). Control of intestinal microbiota by purinergic mediators has been also supported by recent data showing that mice deficient in the ATP-gated ionotropic P2X7 receptor display intestinal microbiotic imbalance and altered glucose metabolism (35).

### Inflammatory Bowel Disease

Inflammatory bowel disease (IBD) is a chronic, debilitating illness characterized by excessive inflammation of the colon and small intestine that is associated with thrombophilia and heightened risk for cancer (36, 37).

Experimental and clinical evidences indicate a protective role of ENTPD1/CD39 in Crohn's disease (CD). Global *Entpd1/Cd39* deletion in dextran-sulfate-sodium (DSS)-induced colitis in mice increases susceptibility to injury (38). Accordingly, high ENTPD1/CD39 expression by circulating Tregs correlates with clinical remission in IBD patients while single nucleotide polymorphisms, associated with low *Entpd1/Cd39* mRNA levels, increase predisposition to Crohn's disease (39).

Crohn's patients have decreased suppressor (sup)Th17-cells, a unique effector cell subtype endowed with immunosuppressive functions. In contrast to conventional pathogenic Th17-cells, supTh17-cells express higher levels of ENTPD1/CD39 (33), more effectively generate eAMP and adenosine and hence can also potently suppress effector T-cell responses via A2A receptors.

Expression of ENTPD1/CD39 can be induced upon engagement of AhR, a mediator of toxin responses and adaptive immunity (40, 41). AhR activation induces accumulation of CD39^+^ and granzyme^+^ human Tregs *in vitro* and treatment with the AhR agonist 2-(1′H-indole-3′- carbonyl)-thiazole-4-carboxylic acid methyl ester has a protective effect in colitic humanized mice by increasing Foxp3^+^, CD39^+^, granzyme B^+^, and IL-10^+^ Tregs (42).

We have recently reported that *in vitro* exposure to unconjugated bilirubin (UCB), a product of heme oxidation that serves as AhR endogenous ligand, results in increased levels of ENTPD1/CD39 and FOXP3 in Th17-cells derived from healthy individuals but not from Crohn's disease patients (18). We have also noted that UCB treatment ameliorates DSS colitis in mice, this protective effect being dependent on ENTPD1/CD39 and AhR (18). Resistance of Crohn's-derived Th17-cells to AhR stimulation results from altered response to hypoxia that inhibits AhR signaling in IBD through induction of ABC transporters; these promoting UCB efflux out of Th17-cells (43).

Furthermore, co-expression of ENTPD1/CD39 and CD161 by T-cells supports Th17 effector phenotype through alterations in both extracellular nucleotide-mediated responses and acid sphingomyelinase catalytic bioactivity that promote IL-17 expression (44, 45). Pro-inflammatory CD4^+^CD39^+^CD161^+^ T-cells are increased in the blood and lamina propria of Crohn's disease patients and levels directly correlate with the disease activity (44). CD3/CD28-mediated stimulation of IFN-γ-producing CD8^+^ T-cells, another effector subset involved in Crohn's disease pathogenesis (46), not only increases IFN-γ production by CD8 T-cells, but also induces reactive oxygen species and ENTPD1/CD39 expression (47).

Increase in CD73^+^CD4^+^ T-cells, which are enriched in IL-17 producing lymphocytes, is detected in the lamina propria and peripheral blood of IBD patients during active inflammation. In Crohn's disease, accumulation of pathogenic Th17-cells has been also associated with heightened CD73 levels (48). Interestingly, exposure to TNF increases CD73 expression on CD4^+^ T-cells, while anti-TNF monoclonal antibody (infliximab) has the opposite effects, therefore implicating CD73^+^ Th17-cells as a surrogate marker of disease activity and response to treatment (48).

Recent data have shown a protective role for ENTPD2 and ENTPD3 in neuro-immune interactions in Crohn's disease (49). ENTPD2-3 are expressed by enteric nervous system cells in both the human and murine colon. Both *Entpd2*^−/−^ and *Entpd3*^−/−^ mice are more susceptible to DSS-induced colitis and *Entpd2*^−/−^ colonic macrophages display a more pro-inflammatory phenotype as compared to wild type controls (49). A significant proportion of the microparticle-associated ectonucleotidase activity is sensitive to POM6, inferring the presence of NTPDases, either −2 or/and −3. Further, human plasma samples obtained from Crohn's patients, show overall decreases in ADPase activity, this alteration being directly correlated with disease activity (49).

Celiac disease is a chronic inflammatory disorder frequently associated with IBD and triggered by aberrant immune responses to dietary gluten. Recent work has shown that gluten exposure induces protective accumulation of FOXP3^+^CD39^+^ Tregs in celiac patients. These Tregs however, are dysfunctional and exhibit impaired suppression (50).

There is evidence that microparticles (MPs) released from cells in the inflammatory site can be loaded with ENTPDase mRNA (51). Such mRNA content within MPs can be taken up by incorporating cells and be subsequently translated into functional NTPDases. This phenomenon occurs between leukocytes and vascular endothelial cells (51). Our collaborators, others and we have also noted that levels of microRNAs (miRs) present in plasma MPs are modulated by CD39 expression and that one microRNA, miR-142-3p, might impacts CD39 levels *per se* (52, 53).

Based on this evidence, MPs could serve as biomarkers of inflammatory pathways as well as therapeutic tools to modulate the function of cells taking up these exosomes and MPs (51–54).

### Colorectal Cancer

Colorectal cancer (CRC) is the second leading cause of tumor-related death in the United States. ENTPD1/CD39 and CD73 are the major ectonucleotidases expressed by tumor endothelial cells and Tregs. Extra-cellular adenosine generated by circulating CD39^+^ Tregs of cancer patients not only inhibits anti-tumor responses and stimulates vascular endothelial cell proliferation, but also reduces monocyte ability to activate the endothelium, limiting migration of effector T-cells into the tumor (55–58).

In a murine model of hepatic metastatic cancer, resulting from portal vein infusion of MCA38 colon cancer cells and melanoma B16/F10 cells, ENTPD1/CD39 expression on Tregs strongly suppresses natural killer (NK)-mediated anti-tumor immunity (59); whereas ENTPD1/CD39 inhibition restores anti-cancer responses, significantly limiting tumor growth (58, 59). The correlation between levels of ENTPD1/CD39 in the host and CRC progression has been confirmed in orthotopic transplanted murine cancer models; while in clinical samples, lower levels of *ENTPD1/CD39* mRNA in malignant CRC tissues correlates with prolonged survival and less invasiveness (60).

The tumor microenvironment strongly impacts the phenotype and function of immune cells, substantially limiting immunotherapeutic strategies. In this regard, most of the CRC-infiltrating Tregs are Helios^+^ and express high levels of ENTPD1/CD39 and cytotoxic T-lymphocyte antigen4 (CTLA-4).

There is also evidence that CD8^+^ lymphocytes infiltrating human CRC recognize a wide range of epitopes unrelated to the tumor, including those recognized during previous viral infections. Such CD8^+^ lymphocytes display wide variability in ENTPD1/CD39 expression, which correlates with the clinical status of patients (61).

## Ectonucleotidases in Acute and Chronic Liver Diseases

Ectonucleotidases can be also expressed in the liver in different cell populations, including resident immune cells and endothelial cells. As observed in the rat, the specific cellular localization and function are strongly affected by variations in the organ homeostasis. In healthy rat liver, CD73 expression partially overlaps with that of ENTPD1/CD39 in fibroblastic cells underneath vascular endothelial cells and smooth muscle cells, and with that of ENTPDase8 in bile canaliculi. In portal spaces, CD73 is expressed in a fibroblast subpopulation, which is adjacent to ENTPDase2^+^ portal fibroblasts. At variance with healthy, quiescent states, the expression, and activity of these ectonucleotidases are largely altered in fibrotic livers (62).

**Figure 2:**
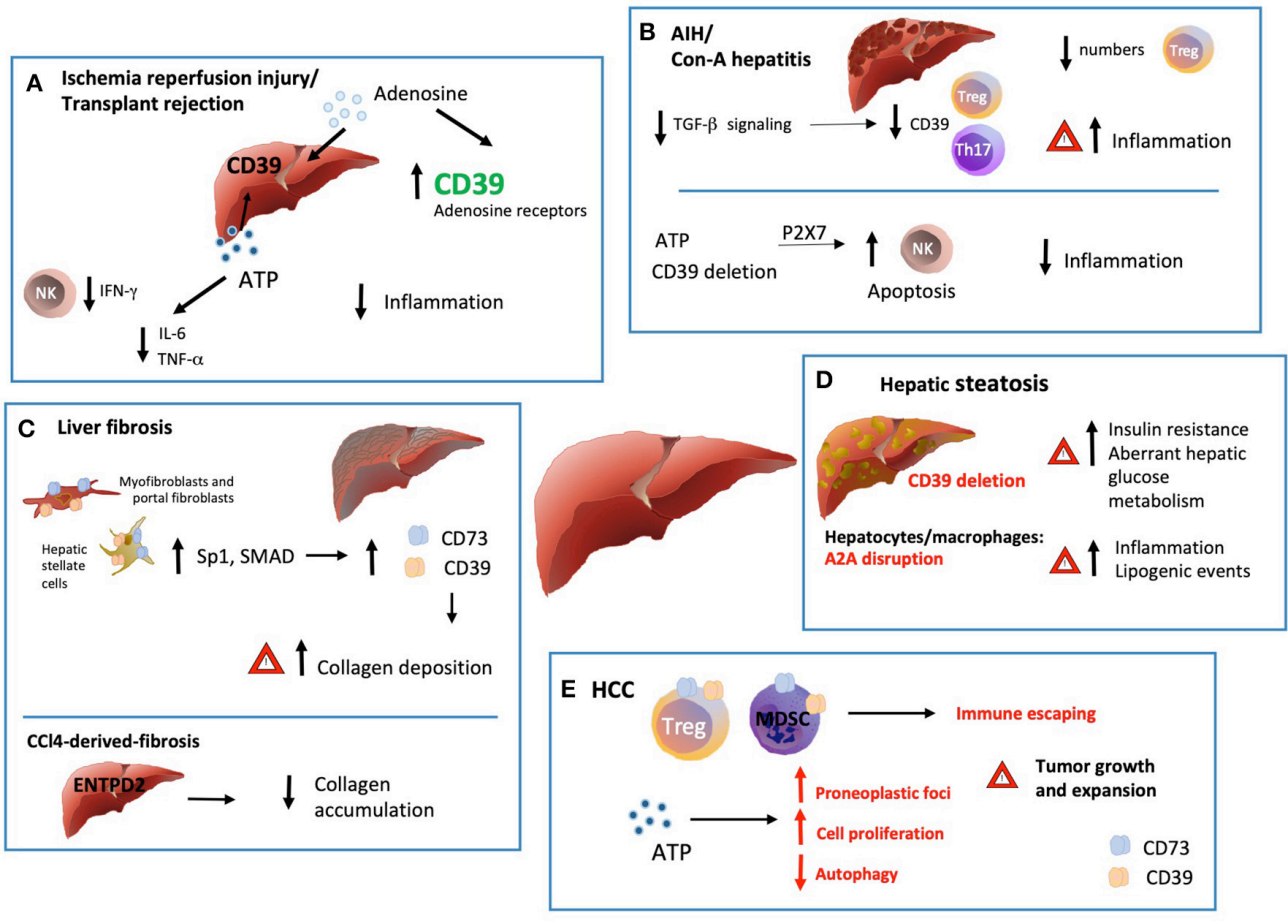
Ectonucleotidases in inflammatory liver conditions. **(A)** Ischemia/reperfusion injury (IRI) is triggered by the vascular damage consequent to blood reperfusion of oxygen deprived ischemic tissues, as with organ transplantation. ENTPD1 expression by donor livers and treatment with exogenous adenosine at high concentrations in preservation solutions protects grafts from ischemic damage with extended cold preservation times. Pharmacologic preconditioning through stimulation of adenosine receptors has been also associated with protection from ischemia by increasing ENTPD1/CD39 expression, via Sp1 transcription factor activation. This protective effect is abrogated in the absence of ENTPD1/CD39 but can be restored by adenosine administration. There is also evidence that exogenous and cautious ATP infusions can improve the hepatic function and post-ischemic clinical condition, (at least in part) by decreasing the plasma levels of IL-6 and TNF. **(B)** Numerical and functional impairment of Tregs contributes to immune imbalance in autoimmune hepatitis (AIH). Tregs and Th17-cells isolated from AIH patients display defective ENTPD1/CD39 expression and fail to control eATP mediated pro-inflammatory Th17 accumulation. Treg acquisition of pro-inflammatory properties together with low ENTPD1/CD39 expression might result from dysfunction in TGF-β signaling. **(C)** Liver fibrosis is driven by activation and accumulation of myofibroblasts and hepatic stellate cells (HSC), the predominant source of extracellular matrix and collagen in the organ. ENTPD1/CD39 and CD73, are upregulated in HSC, portal fibroblasts and in fibrous septa. This overexpression, mediated by SP1 and SMAD promoter elements, is a direct consequence of the myofibroblastic differentiation. Interestingly, in the setting of CCl4-derived-fibrosis, ENTPD2 expression and re-distribution from the portal areas to the fibrotic septa, has a protective role against excessive collagen accumulation. **(D)** In hepatic steatosis and alcoholic hepatitis, *Entpd1/Cd39* deletion correlates with increased insulin resistance and aberrant hepatic glucose metabolism. Accordingly, disruption of A2AR expression in hepatocytes and macrophages also directly correlates with the severity of obesity–associated non–alcoholic-fatty-liver-disease, promoting inflammation and lipogenic events. **(E)** ATP scavenging by ENTPD1/CD39 expressed by CD4^+^FOXP3^+^ Tregs, endothelial cells and myeloid derived suppressor cells (MDSC) promotes hepatic tumor growth in mice. Once exposed to hypoxic microenvironment, HCC upregulates ENTPD2 expression, further supporting MDSC accumulation and immunosuppressive activity. On the other hand, recent experiments have documented occurrence of liver cancer also in *Entpd1/Cd39*^−/−^ mice. These latter findings would result from eATP-P2 receptor-mediated suppression of tumor cell autophagy and boosting of cell proliferation.

Below we discuss the role of ectonucleotidases, especially that of ENTPD1/CD39, in major pathological hepatic conditions (Figure 2).

### Acute Liver Injury

#### Ischemia Reperfusion

Ischemia/reperfusion injury (IRI) is triggered by the vascular damage consequent to blood reperfusion of oxygen deprived tissues. IRI is driven by accumulation of inflammatory mediators, including adenine nucleotides and is associated with platelet activation and, ultimately, organ rejection (63). ENTPD1 expression by donor livers and treatment with high concentration adenosine protect grafts from ischemic damage (64). Pharmacologic preconditioning through stimulation of adenosine receptors also protects from ischemia (65, 66) by increasing ENTPD1/CD39 expression via Sp1 (66). This protective effect, abrogated in *Entpd1/Cd39*^−/−^ mice, can be restored in hemizygous *Cd39*-deficient mice following apyrase or adenosine administration.

ENTPD1/CD39 expression reduces pro-inflammatory activity and promotes protective phenotypes in conventional liver myeloid dendritic cells (mDC) in IRI and transplant models (67, 68). However, there is also evidence that ATP infusion improves hepatic function and post ischemic clinical condition by downregulating IL-6 and TNF plasma levels (69). Similar anti-inflammatory effects are achieved by specifically deleting ENTPD1/CD39 in NK cells, suggesting a regulatory role for ATP/P2 receptor axis during liver injury and subsequent regeneration (70). These results clearly show that, although adenosine and ENTPD1/CD39 are commonly known as immunosuppressive factors, the mechanisms regulating the inflammatory response are complex and markedly impacted by specific cellular conditions.

#### Sepsis

Recent studies have revealed that eATP scavenging has protective effects in sepsis-induced liver injury (71). ENTPD1/CD39 expression by macrophages strongly suppresses pro-inflammatory responses, especially those P2X7-mediated. Accordingly, in the same experimental settings, *Entpd1/Cd39* genetic deletion exacerbates end-organ injury (72).

#### Toxins

Inflammatory liver injury caused by acetaminophen (APAP) toxicity can be linked to purinergic stimulation of immune cells and vascular endothelium. Indeed, P2X7 is crucial in these responses as exposure to ATP ligands is required for manifestations of APAP-induced hepatotoxicity. APAP toxicity is very pronounced in *Entpd1/Cd39*^−/−^ mice, which show hepatic hemorrhagic necrosis and high mortality. Exogenous apyrase also decreases APAP-induced mortality in wild type mice (73).

### Chronic Conditions

#### Autoimmune Hepatitis

Autoimmune hepatitis (AIH) is a severe hepatopathy mediated by aberrant activation of CD8^+^ and CD4^+^ effectors, including Th17-cells. Decreased numbers and functional impairment of Tregs contribute to immune imbalance in AIH (74–76). Tregs isolated from AIH patients display decreased ENTPD1/CD39 expression and fail to control eATP-mediated Th17 accumulation (77). Low levels of and functional defects in Th17CD39^+^ cells have been also detected in juvenile autoimmune liver disease. Here too, the impairments in ENTPD1/CD39 and A2A expression might bolster and promote cellular effector properties. Moreover, levels of adenosine deaminase are significantly increased in AIH patients and positively correlate with inflammation and fibrosis scores (78).

Natural killer T-cells (NKT) are another cell population involved in AIH pathogenesis. In murine models of Concanavalin-A induced hepatitis, genetic deletion of *Entpd1/Cd39* promotes eATP/P2X7-mediated NKT apoptosis and paradoxically provides protection from liver injury (79). Extracellular purines differentially impact different cell types (Treg vs. NKT cells) in certain pathological conditions, as previously shown in the context of hyperoxic lung injury (80). These counter intuitive findings indicate the complexity of purinergic immunomodulation in the liver, and elsewhere (80).

#### Liver Fibrosis

Hepatic fibrosis is a pathological process that develops as a response to chronic inflammation and ongoing liver injury. The pathological process is driven by activation and accumulation of myofibroblasts, a heterogeneous population of activated non-parenchymal liver cells and hepatic stellate cells (HSC). These two cell types are likely the predominant source of extracellular matrix and collagen in the liver.

Different ectonucleotidases, including CD73 and ENTPD family members, are upregulated in HSC, portal fibroblasts and in fibrous septa (62, 81, 82). Such levels of overexpression, mediated by SP1 and SMAD promoter elements, are thought to result from myofibroblastic differentiation.

In experimental models, *Cd73*-deficient mice are resistant to development of liver fibrosis, suggesting a pathological role for AMPase activity and adenosine generation in fibrogenesis (82).

To the contrary, ENTPD2 ATPase activity as expressed by myofibroblasts is protective in the setting of CCl_4_-induced fibrosis as null mice develop more liver scarring in this model. ENTPD2 expression and re-distribution from the portal areas to the fibrotic septa, has a protective role against excessive collagen accumulation in the liver. These salutary effects could be ascribed to anti-inflammatory effects of extracellular ATP scavenging by members of the ENTPD family. In contrast, after partial hepatectomy or 3,5-diethoxycarbonyl-1,4- dihydrocollidine (DDC)-induced hepatocellular injury, the *Entpd2* deletion does not significantly impact the fibrotic response in mutant mice (83).

There is evidence that ENTPD1/CD39 limits hepatic accumulation of gut primed CD8 T-cells, preventing biliary injury and subsequent fibrosis (84). In this context, *Entpd1/Cd39* deletion results in increased levels of hepatic CD8 T-cells following upregulation of the T-cell gut-tropism receptor, integrin α4β7. Accordingly, in *Mdr2*^−/−^*Cd39*^−/−^ mice, CD8 cell depletion as well as gut decontamination and administration of stable ATP agonist or antibiotics, attenuates hepatobiliary injury and fibrosis (47, 83).

#### Hepatic Steatosis/Alcoholic Hepatitis

Purinergic signaling and adenosinergic effects are important modulators of metabolic disease. *Entpd1/Cd39* deletion correlates with increased insulin resistance and aberrant hepatic glucose metabolism (85). Furthermore, A1 adenosine receptor expression on adipocytes impacts fatty acids metabolism, including lipolysis, diabetes, dyslipidemia, and insulin resistance (86).

Adenosine can be also generated during ethanol metabolism and the effects of ethanol-induced hepatic steatosis might be therefore mediated by adenosine receptors, especially A1 and A2B (87). Disruption of A2AR in hepatocytes and macrophages is directly linked to the severity of obesity-associated non-alcoholic-fatty-liver-disease, promoting inflammation and lipogenic events (88).

#### Liver Transplant Rejection

A potentially fatal consequence of liver transplantation is that of immune-mediated organ rejection. Increasing evidences reveal a protective role of *ENTPD1/CD39*, the upregulation and augmented activity of which, achieved also upon exogenous administration, improve liver survival in allotransplantation models (89). Further, ENTPD1/CD39 expression in liver allografts modulates the anti-donor effector T-cell responses and Treg infiltration, ameliorating organ rejection and preventing graft-vs.-host reactions (90).

Liver xenograft rejection is a consequence of vascular inflammation and thrombosis that is partially mediated by extracellular nucleotides (91, 92).

#### Hepatocellular Carcinoma and Metastatic Liver Tumors

Hepatocellular carcinoma (HCC) is the most frequent type of primary liver cancer in adults and is the major cause of death in cirrhotic patients (93). HCC growth and expansion are supported by accumulation of cellular and inflammatory metabolites, including eATP that promotes the generation of preneoplastic foci via P2 receptors (94–96). Further experimentation suggests the development of autochthonous liver cancer in *Entpd1/Cd39*^−/−^ mice (97), resulting from comparable eATP-P2 receptor-mediated changes: inclusive of suppression of liver cell autophagy, altered metabolism, and boosting of proliferation.

Recent work has shown that perturbations in purinergic signaling promote HCC growth, also by supporting immune escaping. ATP scavenging by ENTPD1/CD39 expressed by Tregs and endothelial cells hence facilitates metastatic and transplanted hepatic tumor growth in mice (59, 98, 99).

Generation of adenosine by ENTPD1/CD39 expressed by Tregs and myeloid derived suppressor cells (MDSC) inhibits effector cell proliferation and function (59). Interestingly, in the presence of hypoxic microenvironment, HCC cells upregulate ENTPD2 that preferentially converts extracellular ATP to ADP and little AMP, further supporting the accumulation and immune suppressive activity of MDSC (100).

ENTPD5/CD39L4, a soluble endoplasmic reticulum UDPase can also directly modulate tumor growth impacting N-glycosylation and cell metabolism and has been proposed as target for anti-cancer therapy (53). Other studies, however, have reported contrasting findings, showing increased risk of HCC in *Entpd5* null mice (101).

Table 1 summarizes changes in ectonucleotidase expression and activity in GI and liver experimental models and human conditions.

**Table 1:** Ectonucleotidase expression and activity in GI and liver experimental models and human diseases.

	**Condition**	**Source**	**Ectonucleotidase**	**Function**	**References**
GI	*H. felis*-induced gastritis (mouse)	CD4^+^CD25^+^Foxp3^+^	ENTPD1/CD39 and NT5E/CD73	Protection from excessive inflammation	(28)
	*T. gondii* acute infection (mouse)	CD4^+^ Foxp3^−^ or CD4^+^ Foxp3^+^ T-cells	NT5E/CD73	Downregulation correlates with intestinal immunopathology during lethal infection	(30)
	SIV (mouse) HIV (human)	FOXP3^+^CD25^+^CD4^+^, FOXP3^+^CD25^+^CD8^+^ T-cells	ENTPD1/CD39	Limits anti-viral responses by suppressing the proliferation of SIV/HIV-specific T-cells	(31, 32)
	*Citrobacter rodentium* infection (mouse)	Epithelial cells of small intestine	ENTPD7	Regulation of Th17 cell responses to the pathogen	(34)
	Crohn's disease (human) DSS-induced colitis (mouse)	CD4^+^IL-17^+^CD25^+^ FOXP3^+^, CD4^+^CD25^+^CD127^lo^FoxP3^+^	ENTPD1/CD39	Single nucleotide polymorphisms associated with low *ENTPD1/CD39* mRNA levels and with increased predisposition to Crohn's disease (human); *Entpd1/Cd39* global deletion correlates with disease activity index (mouse)	(18, 33, 38, 39, 43, 50, 51)
		CD4^+^CD25^+^CD127^lo^FoxP3^+^, CD4^+^IL-17^+^IL-10^+^		Protection from tissue damage Global deletion increases disease susceptibility (mouse)	(18, 38, 39)
		CD4^+^ T-cells, CD8^+^ T-cells, CD4^+^CD39^+^CD161^+^ T-cells	NT5E/CD73	Marker of disease activity and response to treatment	(45–48)
		Enteric nervous system	ENTPD2/3	Modulation of neuro-immune interactions and inflammation	(49)
	Colorectal cancer (human, mouse)	PBMCs, CD8^+^ T-cells	ENTPD1/CD39	Low ENTPD1/CD39 expression correlates with prolonged survival and decreased tumor invasiveness	(59, 60)
Liver	Ischemia/reperfusion injury (human, mouse)	CD11b^+^CD11c^+^NK1. 1^−^mPDCA-1^−^ (mDC), global expression on donor and graft tissue cells	ENTPD1/CD39	Protection from ischemic injury Induction of protective anti-inflammatory phenotype	(66, 67)
		NK1.1^+^, CD49b^+^, CD3^−^	ENTPD1/CD39	*Entpd1/Cd39* deletion has an anti-inflammatory effect mediated by ATP/P2X7 toxicity	(69)
	Sepsis	MyD88^+^ macrophages	ENTPD1/CD39	Protection from inflammation-derived organ injury	(72)
	Acetaminophen-induced liver toxicity	CD45.2^+^F480^+^ cells	ENTPD1/CD39	Protection from organ toxicity	(73)
	AIH (human, mouse)	CD4^+^CD25^+^FOXP3^+^ T-cells (human)	ENTPD1/CD39	Immune regulatory properties	(74–77, 79)
				Global deletion causes P2X7-mediated NKT cell apoptosis and protection from ConA-mediated liver injury	
	Liver fibrosis (mouse, rat)	Portal fibroblasts and fibrous septa, hepatic stellate cells	CD73	Induction of fibrotic process	(62, 81, 82)
		Portal fibroblasts and myofibroblasts	ENTPD2	Protection in CCl_4_-induced murine model but not in the DDC-induced model	(83)
		Gut primed-CD8^+^ T-cells	ENTPD1/CD39	Limits accumulation of gut-primed T-cells preventing biliary injury and fibrosis	(84)
	Hepatic steatosis/alcoholic hepatitis (mouse)	Global expression	ENTPD1/CD39	Protection from aberrant hepatic glucose metabolism and insulin resistance	(85)
	Liver transplant rejection (mouse)	Host and liver allograft expression, CD3^+^ CD4^+^ T-cells, CD8^+^ T-cells	ENTPD1/CD39	ENTPD1/CD39 expression in liver allografts modulates graft survival, anti-donor T-cell responses and Treg cell infiltration	(90)
	HCC (mouse, human)	Global expression, CD4^+^CD25^+^Foxp3^+^, CD11b^+^Ly6G^+^Ly6C^+^, CD31^+^ cells	ENTPD1/CD39	Promotes cancer immune escape	(58, 97, 98)
		Tumor (HCC) cells	ENTPD2	Promotes MDSC accumulation	(100)
		Soluble ectonucleotidase, global expression	ENTPD5/CD39L4	Involvement in tumor growth	(53, 101)
	Hepatic metastatic cancer (mouse)	CD4^+^Foxp3^+^ T-cells	ENTPD1/CD39	Suppression of anti-tumor immunity	(58)

*H. felis, Helicobacter felis; T. gondii, Toxoplasma gondii; SIV, simian immunodeficiency virus; HIV, human immunodeficiency virus; DSS, dextran sulfate sodium; PBMCs, peripheral blood mononuclear cells; mDC, myeloid dendritic cells; AIH, autoimmune hepatitis; ConA, concanavalin A; DDC, 3,5-diethoxycarbonyl-1,4- dihydrocollidine; HCC, hepatocellular carcinoma; MDSC, myeloid derived suppressor cells*.

## Concluding remarks

We have briefly discussed how the balance between ATP and adenosine dictates outcomes of inflammatory conditions of the GI tract and liver. Multiple questions, however, remain as to how this balance is regulated, and can be possibly targeted in different disease settings.

Development of purinergic-based therapies that could be used alone or in combination with already existing treatments, might be implemented to control these responses in the gut and liver. The goal of such interventions will be to re-establish and maintain immunologic tolerance and promote healing in these important acute and chronic inflammatory processes.

## Author Contributions

MV wrote the manuscript. SCR and MSL reviewed and edited the manuscript.

### Conflict of Interest Statement

The authors declare that the research was conducted in the absence of any commercial or financial relationships that could be construed as a potential conflict of interest.
